# Evaluation of Ropivacaine and 3-OH-Ropivacaine Pharmacokinetics Following Interpectoral Nerve Block via LC-MS/MS—A Pilot Study

**DOI:** 10.3390/ijms26146696

**Published:** 2025-07-12

**Authors:** Mihaela Butiulca, Lenard Farczadi, Silvia Imre, Camil Eugen Vari, Laurian Vlase, Leonard Azamfirei, Alexandra Elena Lazar

**Affiliations:** 1 Department of Anaesthesiology and Intensive Care Medicine, Faculty of General Medicine, George Emil Palade University of Medicine, Pharmacy, Science, and Technology of Targu Mures, 540142 Targu Mures, Romania; mihaela.budrescu@umfst.ro (M.B.); leonard.azamfirei@gmail.com (L.A.); alexandralazar7@gmail.com (A.E.L.); 2 Department of Anaesthesiology and Intensive Care Medicine, Emergency County Hospital, 540136 Targu Mures, Romania; 3 Chromatography and Mass Spectrometry Laboratory, Center for Advanced Medical and Pharmaceutical Research, George Emil Palade University of Medicine, Pharmacy, Science, and Technology of Targu Mures, 540142 Targu Mures, Romania; silvia.imre@umfst.ro; 4 Department of Analytical Chemistry and Drug Analysis, Faculty of Pharmacy, George Emil Palade University of Medicine, Pharmacy, Science, and Technology of Targu Mures, 540142 Targu Mures, Romania; 5 Department of Pharmacology and Clinical Pharmacy, Faculty of Pharmacy, George Emil Palade University of Medicine, Pharmacy, Science, and Technology of Targu Mures, 540142 Targu Mures, Romania; camil.vari@yahoo.fr; 6 Department of Pharmaceutic Technology and Biopharmacy, “Iuliu Hațieganu” University of Medicine and Pharmacy, 400012 Cluj-Napoca, Romania; laurian.vlase@umfcluj.ro

**Keywords:** ropivacaine, 3-OH-ropivacaine, PECS II block, LC-MS/MS, pharmacokinetics, regional anesthesia, bioanalysis

## Abstract

Regional anesthesia techniques such as the ultrasound-guided PECS II (pectoral nerve block) block are increasingly employed to optimize perioperative analgesia while minimizing systemic anesthetic exposure. Ropivacaine is commonly used for its favorable pharmacological profile; however, clinical data on its pharmacokinetics and systemic metabolite behavior following interpectoral administration remain limited. This study aimed to characterize the plasma concentration–time profile of ropivacaine and its main active metabolite, 3-OH-ropivacaine, in patients undergoing interpectoral nerve block, using a validated LC-MS/MS (liquid chromatography coupled with mass spectrometry) method. Venous blood samples were collected from 18 patients at predefined time points (0, 1, 3, 6, and 24 h) following a PECS II block performed with a ropivacaine-lidocaine mixture. Plasma concentrations were quantified via a validated LC-MS/MS protocol in accordance with FDA (Food and Drug Administration) and EMA (European Medicines Agency) guidelines. Pharmacokinetic parameters were derived using non-compartmental analysis. Ropivacaine reached a mean peak plasma concentration (Cmax—maximum concentration) of 167.5 ± 28.3 ng/mL at 1.3 ± 0.2 h (Tmax—maximum time). The metabolite 3-OH-ropivacaine peaked at 124.1 ± 21.4 ng/mL at 2.3 ± 0.3 h. The terminal elimination half-life was 19.4 ± 2.8 h for ropivacaine and 29.2 ± 3.1 h for its metabolite. Plasma levels demonstrated prolonged systemic exposure with predictable pharmacokinetics. The PECS II block using ropivacaine results in sustained systemic levels of both the parent drug and its primary metabolite, supporting its role in prolonged perioperative analgesia. These data provide a pharmacokinetic foundation for personalized regional anesthesia protocols. This strategy facilitates the adaptation of anesthetic protocols to the individual characteristics of each patient, aligning with the principles of personalized medicine, particularly in patients with altered metabolic capacity.

## 1. Introduction

Ensuring patient comfort and safety during the perioperative period remains a fundamental objective of modern medical care, with a substantial influence on clinical outcomes and postoperative recovery. Regional anesthesia, widely employed to alleviate pain and enhance symptom control, offers several advantages over general anesthesia techniques. The advantages include a lower incidence of systemic complications and a more rapid recovery trajectory [1,2]. Among contemporary local anesthetics, ropivacaine has emerged as a preferred agent due to its favorable pharmacological profile [3]. Its long duration of action and reduced toxicological risk—particularly when compared to bupivacaine—make it an attractive choice for minimizing perioperative risks while maintaining clinical efficacy.

Ropivacaine is a long-acting local anesthetic composed of the pure S(-)-enantiomer, a configuration associated with reduced cardiotoxic and neurotoxic effects commonly observed with racemic or alternative agents [4,5]. The compound is metabolized by the cytochrome P450 enzyme system, giving rise to multiple metabolites, some active and some inactive, including 3-OH-ropivacaine (the main active metabolite), 2′,6′-pipecoloxylidide, 4-OH-ropivacaine, and 2-OH-methyl-ropivacaine [6]. Oxidative metabolization of ropivacaine to the 3-hydroxylated active metabolite occurs predominantly via CYP1A2, while the remaining metabolites are obtained through metabolization by CYP3A4 [6].

In both clinical practice and pharmaceutical research, precise analysis of drugs and their metabolites in biological matrices is essential [7]. Bioavailability, commonly assessed through advanced analytical techniques such as high-performance liquid chromatography (HPLC) and mass spectrometry, directly influences evaluations of a drug’s safety and efficacy [8]. These technologies not only support drug development but also provide critical data for personalized treatment strategies, thereby enhancing therapeutic safety and effectiveness [9].

Furthermore, a comprehensive understanding of the interactions between ropivacaine and physiological variables—as well as the influence of comorbid conditions on drug metabolism—can advise on individualized therapeutic decisions [10]. This is particularly relevant in patients with hepatic or renal impairment, where altered metabolism and excretion pathways may compromise pharmacological outcomes [11,12]. Consequently, an integrated approach that combines pharmacokinetic analysis, advanced biomonitoring technologies, and rigorous clinical assessment is required to maximize the clinical benefits of ropivacaine while minimizing associated risks [13,14].

The PECS (Pectoral Nerve Block) technique, especially the PECS II variant, enables targeted deposition of local anesthetics such as ropivacaine in fascial planes, contributing to effective analgesia and prolonged block duration while also influencing systemic absorption patterns [15,16]. Additives like dexamethasone or adrenaline are frequently used to modulate absorption and prolong analgesic effects.

This study aims to describe an advanced methodology for the characterization of ropivacaine plasmatic pharmacokinetics and bioavailability in patients undergoing regional PECS anesthesia as a foundation for optimizing dosing strategies and minimizing systemic toxicity.

## 2. Results

**Figure 1:**
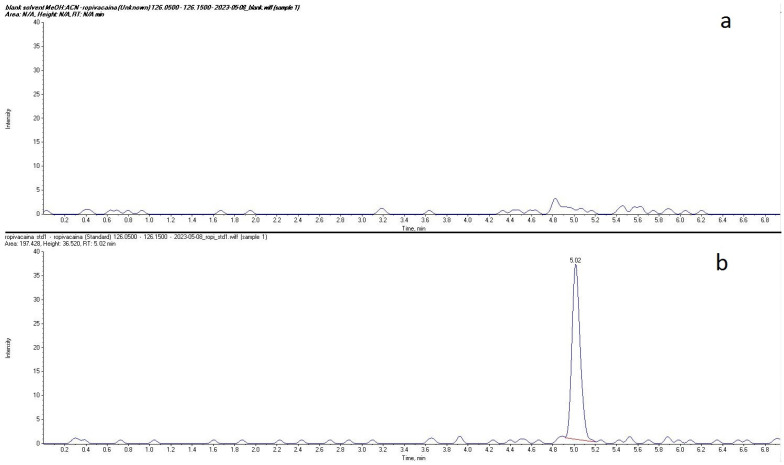
Comparison between extracted ion chromatograms (EIC) for ropivacaine standard solution at the lower limit of quantification (**b**) and blank solvent (**a**).

**Figure 2:**
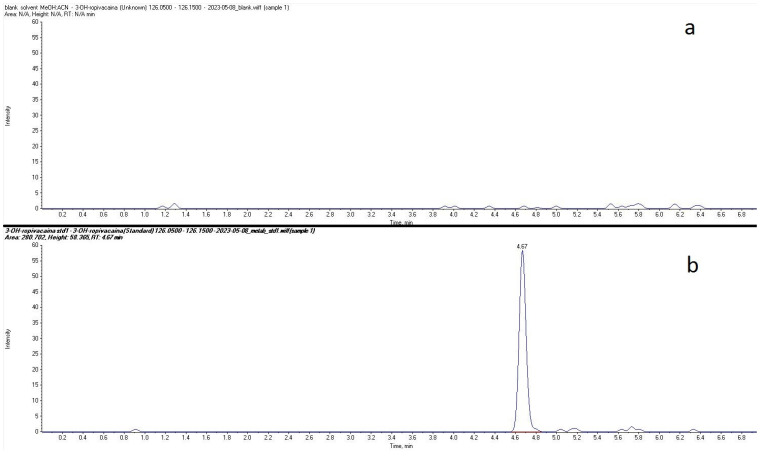
Comparison between extracted ion chromatograms (EIC) for 3-OH-ropivacaine standard solution at the lower limit of quantification (**b**) and blank solvent (**a**).

**Figure 3:**
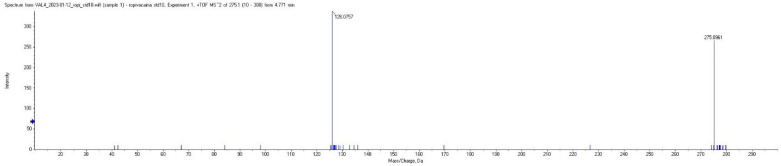
MRM mass spectra for ropivacaine showing parent and fragment ions.

**Figure 4:**
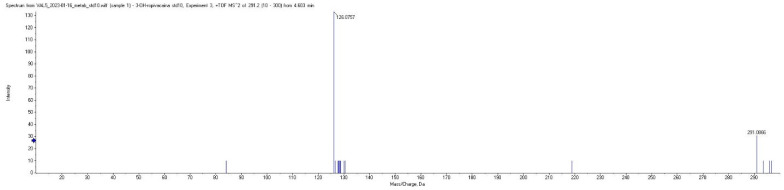
MRM mass spectra for 3-OH-ropivacaine showing parent and fragment ions.

Plasma concentrations of ropivacaine and its primary metabolite, 3-OH-ropivacaine, were quantified using a validated liquid chromatography-tandem mass spectrometry (LC-MS/MS) method—an analytical technique widely recognized for its high sensitivity and specificity, as proven for our method by the lack of interfering peaks when comparing extracted ion chromatograms (XIC/EIC) of ropivacaine standard solutions at the lower limit of quantification to chromatograms of blank solutions (Figure 1 and Figure 2). Ropivacaine and 3-OH-ropivacaine were separated not only chromatographically but also mass spectrometrically based on specific fragmentation patterns for each compound (Figure 3 and Figure 4). Descriptive and non-compartmental pharmacokinetic analyses of the collected data revealed that, on average, the peak plasma concentration of ropivacaine was reached approximately 1.3 h following administration of the interpectoral block. However, most patients exhibited a more rapid absorption profile, occurring at approximately 1 h post-procedure (as summarized in Table 1, Table 2, Table 3 and Table 4).

Following administration, ropivacaine underwent rapid primary metabolism, leading to the formation of 3-OH-ropivacaine. The peak plasma concentration of this metabolite was observed around 2.3 h after the block procedure, indicating a delay of approximately one hour between maximal ropivacaine absorption and peak metabolite concentration. This pharmacokinetic pattern suggests a highly efficient metabolic conversion. This pharmacokinetic pattern is consistent with a rapid and sustained metabolic conversion and a well-defined transition between the absorption and metabolism phases.

After reaching their respective peak concentrations, both ropivacaine and 3-OH-ropivacaine demonstrated gradual elimination from systemic circulation. Ropivacaine exhibited a prolonged plasma half-life exceeding 19 h, indicating slow and continuous elimination. Similarly, 3-OH-ropivacaine displayed an even longer half-life, with systemic persistence observed beyond 29 h post-administration. This extended elimination profile may have significant clinical implications, particularly in patients with impaired hepatic or renal function. This extended elimination profile could influence clinical decisions regarding dosing and monitoring, particularly in patients with impaired hepatic or renal function, where drug metabolism and excretion pathways may be compromised.

**Figure 5:**
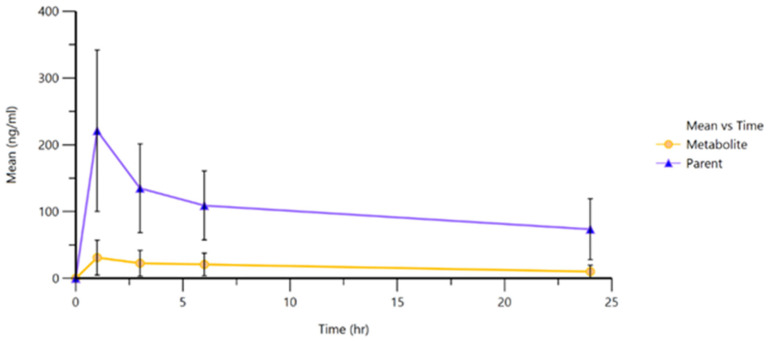
Mean plasmatic concentration curves of ropivacaine and 3-OH-ropivacaine in plasma.

The pharmacokinetic curves (Figure 5) illustrated a clear initial peak in plasma concentration followed by a gradual decline over the subsequent hours within the first day, and it is estimated that the decrease continued over the following days for both ropivacaine and its primary metabolite. This behavior highlights the importance of clinical monitoring and dose individualization, especially in cases where repeated administration or cumulative dosing may pose an increased risk of systemic toxicity.

This detailed analysis enhances our understanding of the pharmacokinetic behavior of ropivacaine, emphasizing both its clinical advantages and the potential risks associated with its use in interpectoral regional blocks.

## 3. Discussion

### 3.1. Pharmacokinetic Considerations

The pharmacokinetic profiles observed in this study confirm the rapid systemic absorption of ropivacaine following interpectoral block administration, with peak plasma concentrations (Cmax) typically occurring around one hour post-injection. This finding is consistent with earlier reports on ropivacaine kinetics in interfascial blocks, particularly those involving thoracic or paravertebral spaces [17,18]. The subsequent formation of 3-OH-ropivacaine, with a time lag of approximately one hour, indicates a relevant metabolic conversion that may contribute to the sustained analgesic effect observed with ropivacaine-based regional techniques.

The prolonged presence of both the parent compound and its metabolite in plasma—reflected by half-lives exceeding 19 and 29 h, respectively—suggests sustained systemic exposure. While this profile may enhance the clinical duration of analgesia, it also raises pharmacological considerations for dose accumulation, especially in patients with compromised hepatic or renal clearance [19].

### 3.2. Clinical and Therapeutic Implications

From a clinical perspective, the systemic kinetics of ropivacaine following PECS block support its application in procedures requiring both immediate and extended pain control, such as cardiac implantable electronic device (CIED) implantation. The metabolization to 3-OH-ropivacaine may extend the analgesic window without necessitating additional anesthetic doses, a desirable feature in elderly or high-risk patients. Comparable kinetic consistency has been reported in continuous PECS blocks and thoracic regional techniques [20].

The extended elimination phase supports the safety and predictability of a single PECS II block but also highlights the need for cautious dosing and monitoring, particularly in patients with reduced drug clearance capacity. Pharmacokinetic-guided dosing may become increasingly relevant in multimorbid populations.

### 3.3. Limitations

This study has several limitations. First, the sample size (*n* = 18) limits the generalizability of the pharmacokinetic results, and the limited timeframe for blood sampling due to the clinical context also limits the amount of pharmacokinetic data obtained. This sample size reflects the exploratory nature of this study and the strict inclusion criteria applied during a defined time. While the limited number of participants reduces the statistical power and constrains the generalizability of the findings, the data provide valuable preliminary insights and may serve as a foundation for future hypothesis generation. The results should therefore be interpreted with caution, and further studies with larger cohorts are warranted to validate the observed trends.

Second, while the prolonged presence of both ropivacaine and its active metabolite in plasma supports the hypothesis of sustained analgesia, it is important to acknowledge that this study did not include clinical pain assessments, such as numerical pain scores or opioid consumption data. As such, although the pharmacokinetic profile suggests potential clinical benefit, the absence of direct pharmacodynamic endpoints limits the ability to draw definitive conclusions about analgesic efficacy. Future studies should aim to correlate systemic exposure with clinical outcomes to validate these findings and guide more tailored perioperative pain management strategies.

Third, although the LC-MS/MS methodology was rigorously validated, the absence of pharmacodynamic data (e.g., pain scores and opioid consumption) prevents direct correlation between systemic concentrations and clinical outcomes. Also, the observational nature of this study, lacking a randomized control group, restricts causal inference.

However, even with these limitations, which are not uncommon in the field of emergency medicine, this study offers valuable insight into the pharmacokinetics of ropivacaine, an area with limited information and data, at the same time offering a great basis for future research directions by offering the first reported pharmacokinetics overview for an entire day (24 h) in patients undergoing regional PECS anesthesia.

### 3.4. Future Directions

Future studies should incorporate larger randomized cohorts to confirm these findings and explore interindividual variability, particularly with respect to cytochrome P450 polymorphisms (e.g., CYP1A2 and CYP3A4) known to affect local anesthetic metabolism. Additionally, integrating pharmacodynamic endpoints and continuous block protocols may further clarify the clinical implications of sustained ropivacaine and metabolite exposure. The development of personalized dosing strategies based on patient metabolic profiles remains a promising avenue for improving anesthetic safety and efficacy.

## 4. Materials and Methods

### 4.1. Selection of Study Subjects

Ethical approval for this study was obtained in accordance with the Declaration of Helsinki from the local Ethics Committees (approval no. 28212/10 November 2021 and no. 1515/9 December 2021). This study was conducted at the Emergency Institute for Cardiovascular Diseases and Transplantation between November 2021 and January 2024.

This study was conducted on a randomly selected sample of 18 patients undergoing de novo implantation of a cardiac device at our institution. Inclusion criteria were age over 18 years, written informed consent to participate in this study, and de novo cardiac device implantation. Exclusion criteria were reimplantation of a cardiac device, inability to communicate effectively (e.g., due to cognitive impairment or language barriers), refusal to participate in this study, and the presence of an active infection at the puncture site.

### 4.2. Anesthetic Procedure for Interpectoral (PECS) Block

**Figure 6:**
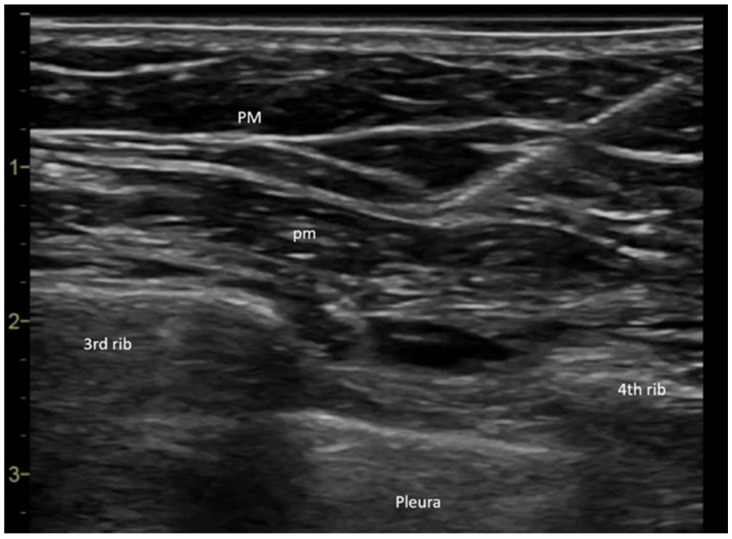
PECS II nerve block (Legend: PM: pectoralis major muscle; pm: pectoralis minor muscle).

All procedures were performed in the operating room by the same anesthesiologist to ensure consistency. Upon arrival, patients received peripheral intravenous access and standard monitoring (heart rate, non-invasive blood pressure, and peripheral oxygen saturation). The PECS block was performed under ultrasound guidance (General Electric Vivid I6, 8L probe)-USA, with patients positioned supine and arm adducted beside the body. The ultrasound transducer was placed medially over the coracoid process and rotated laterally towards the deltopectoral groove to identify the axillary artery. Locating the second and third ribs was crucial for anatomical orientation; the second rib was found immediately beneath the axillary artery, and slight caudal-lateral transducer movement revealed the third rib. The fascial plane between the pectoralis major and minor muscles was targeted for the first injection. Prior to needle insertion, 2 mL of anesthetic mixture was infiltrated using a 27 G needle to minimize pain during puncture with a 20 G plexus needle. Needle advancement was visualized in real-time, and correct placement was confirmed by hydrodissection with saline between the fascial layers. Further caudal-lateral movement of the probe allowed visualization of the fourth rib and the serratus anterior muscle. The second anesthetic injection targeted the fascial plane between the pectoralis minor and the serratus anterior muscles. An echogenic needle (Pajunk Sonoplex 50 mm, 20 G) was used for all regional blocks to improve ultrasound visibility (Figure 6). An equimolar mixture of ropivacaine and 1% lidocaine was administered, with the dose adjusted according to each patient’s ideal body weight. Sensory block efficacy was assessed five minutes post-injection using the cold sensation test.

### 4.3. Sample Collection

To evaluate the plasma concentration and safety profile of ropivacaine, blood samples were collected from a representative cohort of 18 patients who underwent a pectoral nerve block. Blood collection was performed using vacutainer tubes equipped with a clot activator and gel separator. Samples were drawn at predefined time points to capture the pharmacokinetic profile of the drug: before administration of the interpectoral block and subsequently at 1 h, 3 h, 6 h, and 24 h post-procedure. These intervals were selected to characterize the absorption phase, the steady-state plateau, and the drug elimination dynamics.

### 4.4. Sample Processing

Immediately after collecting, the blood samples underwent primary processing. Tubes were centrifuged at 1300× *g* for 10 min to separate the serum from cellular components. The serum was carefully transferred into 1.5 mL Eppendorf tubes, sealed, and stored at −80 °C to preserve compound integrity until further analysis.

Preparation of plasma samples for LC-MS/MS (liquid chromatography coupled with mass spectrometry) analysis involved deproteinization—a critical step for eliminating protein interference and improving measurement accuracy. Deproteinization was achieved by adding 200 μL of plasma to 100 μL of an internal standard solution and 500 μL of acetonitrile, a solvent commonly used for protein precipitation. The resulting mixture was vortexed for two minutes to facilitate thorough interaction, followed by centrifugation at 4000× *g* for three minutes to separate the precipitated protein fraction. The clear supernatant obtained after centrifugation was collected and used directly for LC-MS analysis. The concentration of the internal standard (ropivacaine-d7) in the final sample solutions was 62.5 ng/mL.

The internal standard was rigorously selected to control methodological variability and to ensure accurate quantification of ropivacaine concentrations. Acetonitrile was chosen due to its efficiency in protein precipitation, low toxicity, and cost-effectiveness, facilitating the preparation of high-purity samples for subsequent analysis. Maintaining the samples at −80 °C was critical to preserving the chemical and biological stability of ropivacaine and its metabolites, thereby enabling precise and reproducible analytical results.

### 4.5. Standard Solutions and Analyte Extraction

Stock solutions of ropivacaine and 3-OH-ropivacaine were prepared at concentrations of 500 µg/mL and 100 µg/mL, respectively. These were subsequently diluted to generate working solutions and plasma-based calibration standards. Calibration curves ranged from 0.5 to 1000 ng/mL for ropivacaine and 1 to 1000 ng/mL for 3-OH-ropivacaine.

An internal standard solution of ropivacaine-d7 at a concentration of 0.5 µg/mL in acetonitrile was prepared to ensure methodological consistency. Quality control (QC) samples for ropivacaine were prepared at 2, 30, 500, and 750 ng/mL, while QC samples for 3-OH-ropivacaine were prepared at 3, 30, 400, and 750 ng/mL.

For sample processing, 200 μL of either calibration standards or plasma QC samples were mixed with 100 μL of internal standard solution. Protein precipitation was achieved by adding 500 μL of acetonitrile. The mixture was vortexed for 2 min and centrifuged at 4000× *g* for 3 min. The resulting supernatant was transferred to chromatographic vials and loaded into the autosampler for injection into the LC-MS system.

### 4.6. LC-MS/MS Analysis

The LC-MS system used in this study was specifically optimized for the selective detection of ropivacaine and its metabolites, providing high sensitivity and specificity. This methodological approach enabled the generation of pharmacokinetically meaningful data, crucial for understanding the drug’s in vivo behavior and improving its safety in clinical applications.

Chromatographic separation of ropivacaine, 3-OH-ropivacaine, and ropivacaine-d7 (IS) was performed using a Gemini NX-18, 3.0 × 100 mm (3 μm particles) column (Phenomenex Inc., Torrance, CA, USA), thermostatted at 15 °C, with a mobile phase consisting of 0.05% (*V*/*V*) formic acid in water (mobile phase A) and acetonitrile (mobile phase B) in gradient elution. The mobile phase gradient was as follows: 0–2 min, 95% mobile phase A; 2–4 min, 70% mobile phase A; and 4–7 min, 95% mobile phase A. The flow rate was constant at 0.4 mL/min. Ionization of samples was performed using an electrospray ion source in positive mode, using the following ionization parameters: spray voltage 5000 V, vaporizer temperature 350 °C, source gas1 pressure 35 psi, source gas2 pressure 30 psi, and curtain gas pressure 25 psi. Detection was conducted in multiple reaction monitoring (MRM) mode for ropivacaine, 3-OH-ropivacaine, and ropivacaine-d7 (IS) by monitoring specific fragments for each analyte (Table 5). The total run-time of the method, including the column re-equilibration step, was 7 min per sample. Calibration was compiled using AB Sciex Analyst 1.6 software with the internal standard calibration method, and QC samples were injected together with study samples to ensure measurement accuracy. Although there is a slight mass defect between the detected m/z for ropivacaine and 3-OH-ropivacaine, it is most likely due to a change in the space charge effects of the pseudomolecular ion, as the mass shift can occur even in TOF mass analyzers when higher concentrations of analytes are analyzed. This slight shift in mass is insignificant enough to not influence the measurements, especially since for m/z below m/z 500, mass defects are not considered as crucial as for higher masses, and the method was fully validated beforehand.

The LC-MS/MS method for analyte quantification was validated in compliance with the FDA (Food and Drug Administration) [21] and EMA (European Medicines Agency) [22] bioanalytical method validation guidelines. All key performance parameters—including accuracy, precision, linearity, selectivity, sensitivity, matrix effects, and analyte recovery—were assessed and found to meet regulatory standards. The full development and validation of the method have been previously published [23].

The analytical instrumentation included a Perkin Elmer FX-10 HPLC system (PerkinElmer, Waltham, MA, USA) coupled with an AB Sciex Triple TOF 4600 mass spectrometer (Thermo Fisher Scientific Inc., Waltham, MA, USA). Additional equipment comprised an Eppendorf 5430R centrifuge (Eppendorf, Hamburg, Germany), a Radwag XA 52.3Y analytical balance (Radwag, Radom, Poland), a Velp Scientifica ZX4 vortex mixer (VELP Scientifica Srl, Usmate, Italy), a JP Selecta Ultrasons H-D ultrasonic bath (J.P. Selecta, Abrera, Spain), and a Thermo SpeedVac concentrator-evaporator (Thermo Fisher Scientific Inc., Waltham, MA, USA).

### 4.7. Pharmacokinetic Analysis

Quantification of ropivacaine and its primary metabolite, 3-OH-ropivacaine, was performed using calibration curves with linear regression and 1/y^2^ weighting. Results were expressed in ng/mL of plasma and directly derived from the concentration values of the calibration standards. These measurements formed the basis for subsequent statistical and pharmacokinetic analyses. Plasma concentrations of ropivacaine and 3-OH-ropivacaine were measured using the previously validated LC-MS/MS method, ensuring high reliability and reproducibility of the results.

Initial raw data were processed using Phoenix WinNonlin 7 software for pharmacokinetic evaluation. Both descriptive statistics and pharmacokinetic modeling were conducted, with primary pharmacokinetic parameters calculated using non-compartmental analysis methods. A non-compartmental pharmacokinetic model was chosen for statistical evaluation, as it is the model generally used for single studies and is most often used for establishing the initial exposure characteristics of a drug in preclinical or nonclinical pharmacokinetics, toxicology studies, early clinical research, and other types of bioavailability studies. Using non-compartmental analysis, the most relevant parameters for the pharmacokinetics of ropivacaine were determined, such as maximum plasmatic concentration (Cmax) and time of maximum concentration (Tmax), terminal half-life of the drug (t1/2), area under the concentration-time curve (AUC), volume of distribution (Vd), and systemic clearance (CL).

## 5. Conclusions

Biomonitoring of plasma concentrations of ropivacaine and its active metabolite, 3-OH-ropivacaine, represents a highly valuable tool for precise dose optimization. This strategy facilitates the adaptation of anesthetic protocols to the individual characteristics of each patient, aligning with the principles of personalized medicine. Detailed pharmacokinetic monitoring contributes significantly to minimizing the risk of adverse effects, postoperative complications, and toxicological manifestations associated with anesthetic procedures. By ensuring safe pharmacological profiles and enabling dose individualization, this approach plays a pivotal role in improving clinical outcomes and enhancing the quality of perioperative care.

## Figures and Tables

**Table 1:** Individual non-compartmental pharmacokinetics of ropivacaine.

Probe No.	Cmax (ng·mL^−1^)	Tmax (h)	AUC (hr ∗ ng mL^−1^)	Elimination Rate Constant (1 h^−1^)	Half-Time (h)	Distribution Volume (L kg^−1^)	Clearance (L h^−1^ kg^−1^)
1	537.61	1.00	3037.65	0.05	15.16	9.38	428.54
2	427.72	1.00	5260.61	0.02	29.77	6.60	153.70
3	308.92	1.00	2224.24	0.13	5.45	6.76	859.54
4	169.74	1.00	2270.55	0.03	25.09	14.86	410.45
5	198.65	1.00	2031.03	0.03	20.13	15.89	547.03
6	167.57	6.00	2727.88	0.06	11.04	8.87	557.17
7	216.72	1.00	2781.24	0.04	17.50	11.21	444.09
8	285.97	1.00	2678.63	0.03	20.79	12.53	417.88
9	101.88	1.00	2066.14	0.05	12.81	14.30	773.57
10	293.92	1.00	4887.17	0.02	33.09	7.56	158.39
11	127.46	1.00	1053.57	0.04	18.37	30.23	1141.09
12	297.75	1.00	2778.64	0.11	6.58	6.33	667.60
13	217.28	1.00	1369.15	0.05	15.38	22.30	1005.34
14	106.96	1.00	1704.85	0.06	11.04	15.77	989.71
15	130.01	1.00	1474.43	0.03	22.47	21.46	662.03
16	109.04	1.00	2039.96	0.05	15.38	15.88	715.48
17	101.96	1.00	1646.73	0.02	31.33	21.61	478.04
18	208.90	1.00	2532.91	0.02	35.27	14.89	292.60

Legend: * multiplication sign.

**Table 2:** Individual non-compartmental pharmacokinetics of 3-OH-ropivacaine.

Nr. Ctr.	Cmax (ng mL^−1^)	Tmax (h)	AUC (hr ∗ ng mL^−1^)	Elimination Rate Constant (1 h^−1^)	Half-Time (h)	Volume of Distribution (L kg^−1^)	Clearance (L h^−1^ kg^−1^)
1	35.90	1.00	257.11	0.05	14.53	114.58	5464.18
2	18.71	1.00	219.09	0.00	211.26	109.07	357.87
3	26.07	1.00	264.71	0.08	8.47	79.62	6519.28
4	7.67	6.00	126.07	0.06	10.95	191.71	12,133.02
5	14.50	1.00	167.78	0.03	20.90	192.32	6379.93
6	33.60	1.00	402.42	0.02	36.78	102.89	1939.08
7	8.21	6.00	137.47	0.06	12.49	187.73	10,419.02
8	47.81	1.00	439.43	0.04	16.18	67.22	2878.89
9	3.29	6.00	54.77	0.05	13.42	501.02	25,870.16
10	25.39	1.00	147.85	0.06	12.46	188.99	10,511.42
11	36.07	6.00	548.37	0.08	8.34	37.27	3098.18
12	99.48	1.00	1176.18	0.10	6.83	15.43	1564.44
13	33.23	1.00	207.48	0.28	2.46	43.32	12,194.29
14	38.35	1.00	501.29	0.03	21.18	63.47	2077.53
15	77.63	1.00	1072.98	0.01	61.25	39.53	447.36
16	40.53	3.00	531.15	0.03	27.11	61.58	1574.20
17	27.28	3.00	384.83	0.06	11.72	65.94	3899.52
18	58.42	1.00	620.60	0.02	29.53	58.54	1373.95

**Table 3:** Mean pharmacokinetics of ropivacaine after non-compartmental analysis.

Ropivacaine	Mean	SD	CV (%)	Median	Geometric Mean
Cmax (ng mL^−1^)	222.67	119.60	53.71	203.77	196.93
Tmax (h)	1.28	1.18	92.23	1.00	1.10
AUC (hr ∗ ng mL^−1^)	2475.85	1094.68	44.21	2247.39	2284.99
Elimination rate constant (1 h^−1^)	0.05	0.03	61.91	0.04	0.04
Half-time (h)	19.26	8.85	45.93	17.93	17.16
Volume of distribution (L kg^−1^)	14.25	6.52	45.73	14.58	12.90
Clearance (1 h^−1^ kg^−1^)	594.57	282.71	47.55	552.10	521.32

**Table 4:** Mean pharmacokinetics of 3-OH-ropivacaine after non-compartmental analysis.

3-OH-Ropivacaine	Mean	SD	CV (%)	Median	Geometric Mean
Cmax (ng mL^−1^)	35.12	24.40	69.49	33.41	26.75
Tmax (h)	2.33	2.11	90.62	1.00	1.68
AUC (hr ∗ ng mL^−1^)	414.42	307.58	74.22	393.62	317.65
Elimination rate constant (1 h^−1^)	0.06	0.06	102.72	0.05	0.04
Half-time (h)	29.22	47.45	162.42	13.98	16.90
Volume of distribution (L kg^−1^)	117.79	111.96	95.05	73.42	86.06
Clearance (L h^−1^ kg^−1^)	6039.02	6366.91	105.43	3498.85	3529.67

**Table 5:** Mass spectrometric fragments monitored for analyte quantification.

Analyte	Monoisotopic Molecular Weight (g/mol)	Expected Parent Ion [M + 1] (*m*/*z*)	Observed Parent Ion (*m*/*z*)	Fragment Ion (*m*/*z*)
Ropivacaine	274.2	275.2	275.096	126.076
3-OH-ropivacaine	290.2	291.2	291.087	126.076
Ropivacaine-d7 (internal standard)	281.2	282.2	282.096	133.076

## Data Availability

Data are contained within the article.

## Abbreviations

The following abbreviations are used in this manuscript:

PECS II	Pectoral Nerve Block
LC-MS/MS	liquid chromatography coupled with tandem mass spectrometry
FDA	Food and Drug Administration
EMA	European Medicines Agency
Cmax	maximum plasmatic concentration
Tmax	time of maximum concentration
HPLC	high-performance liquid chromatography
QC	quality control
AUC	area under the curve
SD	standard deviation
CV	coefficient of variation
